# Markers of Myocardial Ischemia in Patients with Obstructive Sleep Apnea and Coronary Artery Disease

**DOI:** 10.1155/2015/621450

**Published:** 2015-05-18

**Authors:** Misa Valo, Annette Wons, Albert Moeller, Claudius Teupe

**Affiliations:** Center of Sleep Medicine, Department of Medicine, Krankenhaus Sachsenhausen, 60594 Frankfurt, Germany

## Abstract

Obstructive sleep apnea (OSA) is characterized by intermittent hypoxia during sleep. We tested the hypothesis that nocturnal myocardial ischemia is detectable by ST segment depression and elevation of high sensitive troponin T (hsTrop T) and B-type natriuretic peptide (NT-proBNP) in patients with OSA and coexisting coronary artery disease (CAD). Twenty-one patients with OSA and CAD and 20 patients with OSA alone underwent in-hospital polysomnography. Blood samples for hsTrop T and NT-proBNP measurements were drawn before and after sleep. ST segment depression was measured at the time of maximum oxygen desaturation during sleep. The apnea-hypopnea-index (AHI), oxygen saturation nadir, and time in bed with oxygen saturation of ≤80% were similar in both groups. Levels of hsTrop T and NT-proBNP did not differ significantly before and after sleep but NT-proBNP levels were significantly higher in patients suffering from OSA and CAD compared to patients with OSA alone. No significant ST depression was found at the time of oxygen saturation nadir in either group. Despite the fact that patients with untreated OSA and coexisting CAD experienced severe nocturnal hypoxemia, we were unable to detect myocardial ischemia or myocyte necrosis based on significant ST segment depression or elevation of hsTrop T and NT-proBNP, respectively.

## 1. Introduction

Obstructive sleep apnea (OSA) is associated with an increased risk of cardiovascular morbidity and mortality [1]. OSA is characterized by intermittent hypoxia during sleep, which is associated with elevated sympathetic activity, cardiovascular variability, and intrathoracic pressure changes. OSA also provokes systemic inflammation, oxidative stress, endothelial dysfunction, insulin resistance, and thrombosis [2]. Atrial fibrillation, coronary artery disease, congestive heart failure, and arterial hypertension are clinical manifestations and are more common in patients with OSA [3, 4]. Stress imposed on the myocardium by repeated severe hypoxemia during sleep and an increased oxygen demand by sympathetic overstimulation in OSA may result in subclinical myocardial injury [5]. Cardiac troponin T is an important biomarker in myocardial injury and a predictor of clinical outcome [6]. Cardiac myocytes constitute the major source of N-terminal pro B-type natriuretic peptide (NT-proBNP). This is secreted by myocytes after myocardial hypoxemia and ventricular volume overload [7]. NT-proBNP production is strongly upregulated in cardiac failure and locally in the area surrounding a myocardial infarction [8].

We hypothesized that moderate to severe OSA may precipitate myocardial ischemia in patients with coexisting CAD, reflected by ST-segment depression. We also evaluate whether repetitive nocturnal hypoxia in these patients may cause low-grade myocardial injury, as demonstrated by increased levels of NT-proBNP and highly sensitive troponin T (hsTrop T).

## 2. Methods

The prospective study was approved by the ethics committee of the State Medical Council of Hessen, Germany (approval number FF 6/2912). Informed consent was obtained from each patient.

### 2.1. Study Population

Consecutive patients were screened by polygraphy for the presence of moderate to severe OSA with a minimum oxygen desaturation of ≤80% during apnea. The study ultimately enrolled a group of 21 patients with untreated OSA and concomitant CAD proven by coronary angiography (group 1) who were referred for complete polysomnography (PSG). A group of 20 patients with untreated OSA but without a history of CAD and free from CAD symptoms served as a control (group 2).

Inclusion criteria were as follows: age >18 years, apnea-hypopnea-index (AHI) ≥15/h, oxygen desaturation ≤80% at PSG, proven history of CAD (group 1), and untreated OSA. Exclusion criteria were heart failure (left ventricular ejection fraction <40% measured by echocardiography) and renal insufficiency (glomerular filtration rate <50 mL/min estimated using the Cockcroft-Gault formula).

### 2.2. Polysomnography

The presence and severity of OSA were determined by overnight complete polysomnography using a computerized system (Alice 5, Philips Respironics, Herrsching, Germany). Standard techniques such as EEG, electrooculography, electromyography, electrocardiogram, thermistor measurements of air flow, thoracoabdominal motion, pulse oximetry of arterial oxyhemoglobin saturation (SPO_2_), and body position were used to monitor sleep disordered breathing. Bedtime was 10 p.m. to 6 a.m. Sleep stages were scored according to the standard criteria of the American Academy of Sleep Medicine [9]. Apnea was defined as an absence of airflow for >10 s. Hypopnea was defined as a more than 30% reduction in airflow accompanied by a decrease in SPO_2_ of >4%. AHI was calculated as the average number of apneas and hypopneas per hour of sleep. An AHI ≥15/h was defined as moderate to severe OSA.

### 2.3. Measurement of hsTrop T and NT-proBNP

Quantitative measurement of hsTrop T was achieved via an immunoassay for the in vitro quantitative determination of cardiac troponin T in human serum and plasma (Cobas e 411 Roche Troponin T hs STAT; Roche Diagnostics Inc.) with the upper limit of normal of 14 pg/mL representing the 99th percentile in a normal reference population and a coefficient of variation of <10%. HsTrop T values below the Limit of Blank are reported as <3 ng/L.

NT-proBNP levels were measured with an immunoassay for the in vitro quantitative determination of NT-proBNP in heparinized venous blood (Cobas Roche Cardiac proBNP+; Roche Diagnostics Inc.). Values below the Limit of Blank are reported as <60 ng/L.

Venous blood samples (5 mL) were collected at different time points: before (9 p.m.) and after (7 a.m.) polysomnography. The measurements were performed immediately after the second set of blood samples were drawn.

### 2.4. ST Segment Analysis in the Electrocardiogram

A continuous ECG recording was performed simultaneously during sleep in all patients to screen for ST segment depression episodes as an indicator of myocardial ischemia. Lead II was used to analyze the ST segment for myocardial ischemia. Additional leads I, III, aVL, aVR, and aVF were used as backup for further evaluation of features suggestive of cardiac ischemia observed in the main ECG lead. An ischemic episode was defined as a horizontal or downsloping ST segment depression of ≥1 mm (100 *μ*V) from baseline, measured 80 ms after the J point. The time point of maximum oxygen desaturation during sleep was determined by continuous oximetry. The analysis of the ST segments was performed 10 cardiac cycles after the oxygen saturation nadir. The ST segments in the next 10 consecutive cardiac cycles were analyzed and averaged.

### 2.5. Statistics

The results are expressed as mean ± standard deviation (SD) for continuous variables. For the descriptive statistics, the arithmetic mean, SD, median, minimum and maximum, and 1st and 3rd quartiles were calculated. For comparison of the two groups, the Fisher exact test and the Wilcoxon-Mann-Whitney *U* test (*U* test) were used. The Wilcoxon Matched Pairs Test was applied for paired samples. Statistical analyses were performed using a statistical software package (BiAS for Windows, Version 10.05). *P* values <0.05 were considered statistically significant.

## 3. Results

The study population included a group of 21 patients with OSA and cooccurring CAD (group 1). Within this group, previous coronary artery bypass graft operation had been performed in 5 patients and percutaneous coronary interventions in 16 patients, and CAD proven by angiogram was treated medically in 2 patients. A group of 20 patients with OSA but with no history of CAD and free from CAD symptoms served as a control (group 2). Anthropometric characteristics and clinical data are presented in Table 1.

There were no statistically significant differences between the two groups other than the presence of hypercholesterolemia, history of myocardial infarction, and medication with ACE-inhibitors and *β*-blockers.

**Figure 1:**
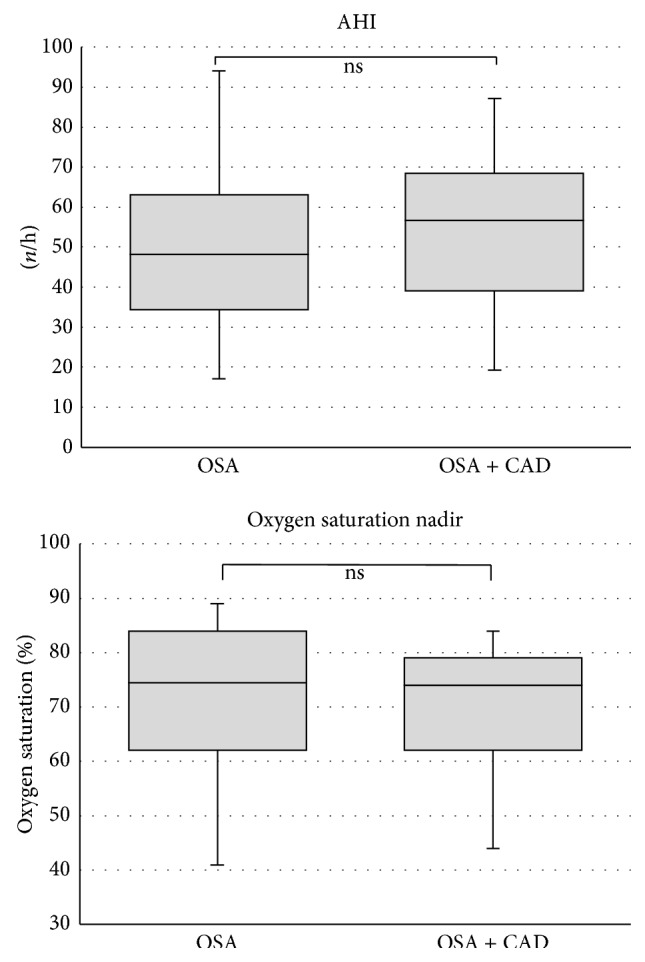
Comparison of polysomnography data (AHI: apnea-hypopnea-index; CAD: coronary artery disease; OSA: obstructive sleep apnea). Middle horizontal line inside box indicates median. Bottom and top of the box are 25th and 75th percentiles, and the error bars outside the box represent maximum and minimum values, respectively.

AHI and oxygen saturation nadir were similar in both groups (Figure 1). Additional polysomnographic variables are presented in Table 2. There were no significant differences between the two groups.

**Figure 2:**
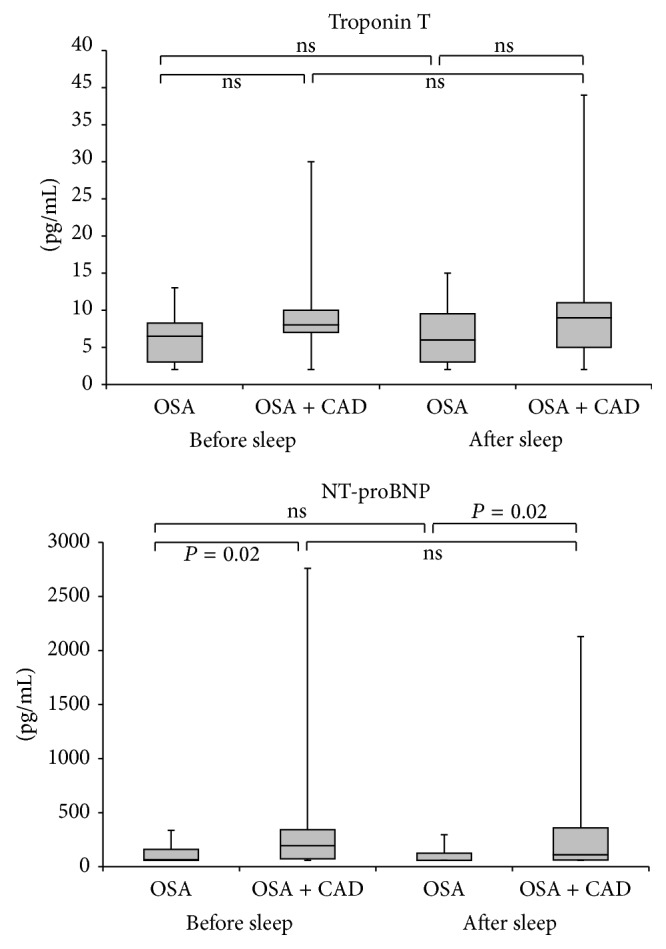
Distribution of troponin T and NT-proBNP levels before and after sleep (CAD: coronary artery disease; OSA: obstructive sleep apnea). Middle horizontal line inside box indicates median. Bottom and top of the box are 25th and 75th percentiles, and the error bars outside the box represent maximum and minimum values, respectively.

The levels and distribution of the cardiac markers hsTrop T and NT-proBNP did not differ significantly before and after sleep within the same group. However, NT-proBNP levels were higher before sleep as well as after sleep in patients with OSA and coexisting CAD compared to patients with OSA alone. HsTrop T was detectable (≥3 ng/L) in 18 (86%) patients before as well as after polysomnography in group 1 and in 16 (80%) patients before and after polysomnography in group 2. Mean hsTrop T levels before and after sleep were 10 ± 7 ng/L and 10 ± 9 ng/L in patients in group 1 and 6 ± 4 ng/L and 7 ± 4 ng/L in group 2, respectively. Mean NT-proBNP levels were 499 ± 758 pg/mL and 400 ± 592 pg/mL before and after sleep in patients in group 1 and 123 ± 93 pg/mL and 101 ± 68 pg/mL in group 2 (Figure 2).

**Figure 3:**
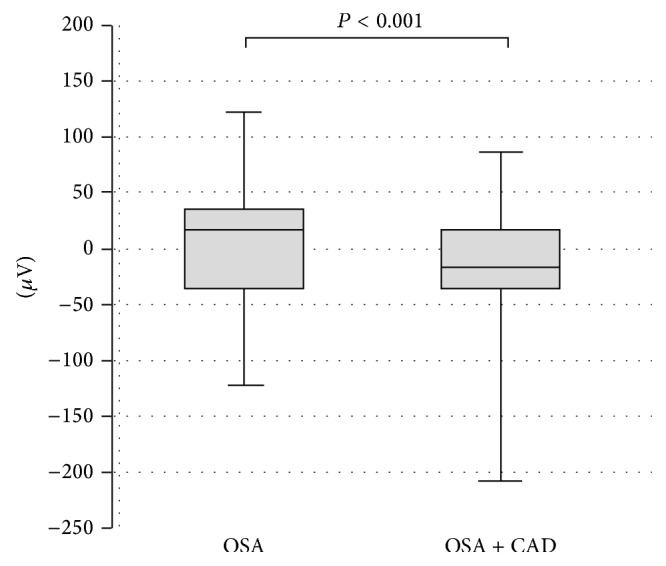
ST segment depression at the time of the deepest oxygen desaturation (CAD: coronary artery disease; OSA: obstructive sleep apnea). Middle horizontal line inside box indicates median. Bottom and top of the box are 25th and 75th percentiles, and the error bars outside the box represent maximum and minimum values, respectively.

ST segment analysis revealed no significant ST depression (≥100 *μ*V) at the time of the deepest oxygen desaturation, neither in patients with OSA and coexisting CAD nor in the control group. However, ST segment depression was significantly more pronounced in group 1 patients than in group 2 patients (Figure 3).

## 4. Discussion

It is well known that OSA is associated with adverse effects on cardiac structure and function. There is a linear relationship between the severity of OSA and patient morbidity and mortality [10]. Cardiac damage in OSA may be caused by activation of the sympathetic nervous system due to hypoxia and changes in negative intrathoracic pressure and increased oxidative stress [11].

Previous studies have suggested that the main risk factor for myocardial ischemia in patients with OSA is inadequate oxygen supply in the presence of CAD, while others assume an increase in oxygen demand due to tachycardia and sympathetic activation following the rebreathing phase after an apnea event [12–14]. The potential cellular mechanism in intermittent hypoxia-induced cardiac damage is an increase in apoptosis and cardiac fibrosis, a decreased arterial vessel and capillary density, and the loss of troponin I [15].

In the current study, we found no evidence of myocardial ischemia or myocyte necrosis in patients with moderate to severe OSA and coexisting CAD based on significant ST segment depression or elevation of hsTrop T. NT-proBNP levels were significantly higher in patients who suffered from OSA and CAD compared to controls but were unchanged after sleep.

The results of previously presented studies which investigated the relation between OSA and myocardial damage measured by different troponins are not univocal. Similar to our findings, a study by Gami et al. reported no myocardial injury detectable by troponin T assay despite the fact that patients with OSA and concomitant CAD experience nocturnal ischemia [16]. In this study, the AHI was comparable to our study but the mean nocturnal oxygen saturation nadir was less distinct (SPO_2_  83 ± 6%). Two other studies found higher levels of hsTrop T and hsTrop I, respectively, independently correlated with OSA severity, suggesting that frequent apneas or hypoxemia in OSA may cause low-grade myocardial injury and play a role in the association between OSA and risk of heart failure [17, 18]. In a study including 505 subjects drawn from the general population, hsTrop T was detectable (≥3 ng/L) in 216 subjects. The proportion of subjects with detectable hsTrop T increased with increasing severity of OSA. But after adjustment for significant univariate predictors of detectable hsTrop T, the association between AHI and hsTrop T was no longer statistically significant [19].

The mechanism of ST segment depression during sleep in patients with OSA is not fully understood. Inspiration against occluded upper airways causing periodic negative changes in intrathoracic pressure and alterations in cardiac preload and afterload may result in myocardial ischemia in the absence of hypoxemia [20].

We found a more pronounced but not significant ST depression of ≥1 mm at the time of oxygen saturation nadir in patients suffering from OSA and CAD. Previous studies have noted ST segment depression in about one-third of OSA patients with CAD predominantly during apneas and reduced oxygen saturation [13, 14]. A study of 226 patients referred for coronary angiogram because of angina pectoris found nocturnal ST segment depression within 2 minutes after apnea-hypopnea or desaturation in only 12% of patients [21]. Apnea-associated ST segment depression was often preceded by a significant increase in heart rate, repetitive apneas, and severe oxygen desaturation. A temporal relationship between sleep-disordered breathing and myocardial ischemia was present only in a minority of patients. Hanly et al. also found asymptomatic ST depression during sleep in 30% of patients with OSA who did not have a history of CAD [22].

We found significantly higher NT-proBNP levels in patients with OSA and coexisting CAD compared to controls. Several studies found higher serum NT-proBNP values in patients with OSA, while other studies found no correlation between OSA severity and NT-proBNP levels [17, 23–26]. NT-proBNP is also an independent predictor for patients with CAD [27]. In patients with stable angina pectoris NT-proBNP serum concentrations showed a close relationship with the extent of CAD and inducible myocardial ischemia [28, 29]. A meta-analysis indicated strong associations between the circulating concentration of NT-proBNP and the long-term prognosis in patients with stable coronary artery disease [30]. NT-proBNP is also strongly associated with mortality in patients with suspected or confirmed unstable CAD [31].

The present study did have some limitations that deserve comment. First is the small number of the study group, which limits interpretation of the results, in particular the *P* values. Second, in patients with OSA alone, a concomitant CAD was excluded only by patient history, although there was a chance that some patients in this group suffered from unrecognized CAD. On the other hand, most patients with coexisting CAD had previous coronary interventions, which may also affect ischemic thresholds. Third, it is possible that the chosen time point for the analysis of ST segment depression after oxygen saturation nadir might not represent the time of most severe myocardial ischemia. ST depression may have been more pronounced during postapnea tachycardia. We also did not take into account the duration of hypoxemia. Other mechanisms like sympathetic activation with increased heart rate and blood pressure were not considered in exploring the relationship between OSA and myocardial injury since most of the patients with OSA and coexisting CAD were on medications (*β*-blockers, ACE inhibitors) which may confound these findings.

## 5. Conclusion

In patients with moderate to severe OSA and coexisting CAD, repeated oxygen desaturation did not result in myocardial necrosis based on elevation of hsTrop T serum levels after sleep. At the time of maximum oxygen desaturation during sleep, we found no significant ST segment depression. Patients with OSA and concomitant CAD had higher NT-proBNP serum levels compared to patients with OSA alone, but NT-proBNP levels were not affected by severe nocturnal hypoxia. Cardiovascular injury in patients with significant OSA seems to be independent of direct ischemia-induced myocardial necrosis even in patients with manifest CAD.

## Figures and Tables

**Table 1:** Clinical characteristics.

	OSAS + CAD (*n* = 21)	OSAS (*n* = 20)	*P* value
Demographics			
Gender (female/male)	5/16	3/17	n.s.
Age (yr)	61 ± 11	54 ± 12	n.s
Body mass index (kg/m^2^)	35 ± 7	33 ± 6	n.s.
Medical history			
Hypertension	19 (90%)	13 (65%)	n.s.
Hypercholesterolemia	17 (81%)	5 (25%)	<0.001
Diabetes mellitus	10 (48%)	5 (25%)	n.s.
Arrhythmia	6 (29%)	1 (5%)	n.s.
Stroke	2 (10%)	0 (0%)	n.s.
Myocardial infarction	12 (57%)	0 (0%)	<0.001
COPD	4 (19%)	2 (10%)	n.s.
Medication			
Nitrates	3 (14%)	0 (0%)	n.s.
*β*-Blockers	19 (90%)	7 (35%)	<0.001
Renin inhibitor	0 (0%)	1 (5%)	n.s.
ACE inhibitors	14 (67%)	3 (15%)	<0.01
Angiotensin II receptor blockers	3 (14%)	5 (25%)	n.s.
Diuretics	11 (52%)	5 (25%)	n.s.
Calcium channel blockers	9 (43%)	5 (25%)	n.s.
*α*-Blockers	1 (5%)	2 (10%)	n.s.
Cardiac and renal function			
LVEF (%)	59 ± 9	65 ± 2	n.s.
GFR (mL/min)	121 ± 53	137 ± 43	n.s.
Polygraphy (screening)			
AHI (*n*/h)	39 ± 18	43 ± 18	n.s.
Oxygen saturation nadir (%)	70 ± 10	71 ± 8	n.s.

Data are presented as mean ± SD or No. ACE: angiotensin-converting enzyme; AHI: apnea-hypopnea-index; GFR: glomerular filtration rate; LVEF: left ventricular ejection fraction.

**Table 2:** Polysomnographic data.

	OSAS + CAD (*n* = 21)	OSAS (*n* = 20)	*P* value
Polysomnography			
AHI (*n*/h)	53 ± 21	49 ± 20	n.s.
Oxygen saturation nadir (%)	71 ± 12	71 ± 15	n.s.
Percentage of time with SaO_2_ <90% (%)	18 ± 17	18 ± 18	n.s.
Percentage of time with SaO_2_ <80% (%)	2 ± 4	4 ± 7	n.s.
Arousal index (*n*/h)	40 ± 17	40 ± 26	n.s.
Desaturation index (*n*/h)	53 ± 29	45 ± 26	n.s.
Maximal duration of SRBD (sec)	89 ± 56	82 ± 23	n.s.

Data are presented as mean ± SD. AHI: apnea-hypopnea-index; SaO_2_: oxygen saturation; SRBD: sleep-related breathing disorder.
